# Depression Status, Lifestyle, and Metabolic Factors With Subsequent Risk for Major Cardiovascular Events: The China Cardiometabolic Disease and Cancer Cohort (4C) Study

**DOI:** 10.3389/fcvm.2022.865063

**Published:** 2022-05-26

**Authors:** Xi Chen, Zhelong Liu, Yan Yang, Gang Chen, Qin Wan, Guijun Qin, Li Yan, Guixia Wang, Yingfen Qin, Zuojie Luo, Xulei Tang, Yanan Huo, Ruying Hu, Zhen Ye, Lixin Shi, Zhengnan Gao, Qing Su, Yiming Mu, Jiajun Zhao, Lulu Chen, Tianshu Zeng, Qiang Li, Feixia Shen, Li Chen, Yinfei Zhang, Youmin Wang, Huacong Deng, Chao Liu, Shengli Wu, Tao Yang, Mian Li, Yu Xu, Min Xu, Tiange Wang, Zhiyun Zhao, Jieli Lu, Yufang Bi, Xuefeng Yu, Weiqing Wang, Guang Ning

**Affiliations:** ^1^Division of Endocrinology, Department of Internal Medicine, Tongji Hospital, Tongji Medical College, Huazhong University of Science and Technology, Wuhan, China; ^2^Branch of National Clinical Research Center for Metabolic Disease, Wuhan, China; ^3^Fujian Provincial Hospital, Fujian Medical University, Fuzhou, China; ^4^The Affiliated Hospital of Luzhou Medical College, Luzhou, China; ^5^The First Affiliated Hospital of Zhengzhou University, Zhengzhou, China; ^6^Sun Yat-sen Memorial Hospital, Sun Yat-sen University, Guangzhou, China; ^7^The First Hospital of Jilin University, Changchun, China; ^8^The First Affiliated Hospital of Guangxi Medical University, Nanning, China; ^9^The First Hospital of Lanzhou University, Lanzhou, China; ^10^Jiangxi Provincial People’s Hospital Affiliated to Nanchang University, Nanchang, China; ^11^Zhejiang Provincial Center for Disease Control and Prevention, Hangzhou, China; ^12^Affiliated Hospital of Guiyang Medical College, Guiyang, China; ^13^Dalian Municipal Central Hospital, Dalian, China; ^14^Xinhua Hospital Affiliated to Shanghai Jiao Tong University School of Medicine, Shanghai, China; ^15^Chinese People’s Liberation Army General Hospital, Beijing, China; ^16^Shandong Provincial Hospital Affiliated to Shandong University, Jinan, China; ^17^Union Hospital, Tongji Medical College, Huazhong University of Science and Technology, Wuhan, China; ^18^The Second Affiliated Hospital of Harbin Medical University, Harbin, China; ^19^The First Affiliated Hospital of Wenzhou Medical University, Wenzhou, China; ^20^Qilu Hospital of Shandong University, Jinan, China; ^21^Central Hospital of Shanghai Jiading District, Shanghai, China; ^22^The First Affiliated Hospital of Anhui Medical University, Hefei, China; ^23^The First Affiliated Hospital of Chongqing Medical University, Chongqing, China; ^24^Jiangsu Province Hospital on Integration of Chinese and Western Medicine, Nanjing, China; ^25^Karamay Municipal People’s Hospital, Xinjiang, China; ^26^The First Affiliated Hospital of Nanjing Medical University, Nanjing, China; ^27^Department of Endocrine and Metabolic Diseases, Shanghai Institute of Endocrine and Metabolic Diseases, Ruijin Hospital, Shanghai Jiao Tong University School of Medicine, Shanghai, China; ^28^Shanghai National Clinical Research Center for Metabolic Diseases, Key Laboratory for Endocrine and Metabolic Diseases of the National Health Commission of the PR China, Shanghai National Center for Translational Medicine, Ruijin Hospital, Shanghai Jiao Tong University School of Medicine, Shanghai, China

**Keywords:** depression, lifestyle risk factor, metabolic risk factor, major cardiovascular event, prospective cohort study

## Abstract

**Background:**

We aimed to evaluate the association between depression and major cardiovascular events and test whether the relationship between depression and cardiovascular events is influenced by lifestyle or metabolic risk factors.

**Methods:**

The China Cardiometabolic Disease and Cancer Cohort (4C) Study was a nationwide, multicenter, prospective cohort study. About 92,869 participants without cardiovascular disease or cancer at baseline were included. Depression status was evaluated by the Patient Health Questionnaire-9 (PHQ-9). Lifestyle information was collected by the questionnaire, and metabolic risk factors including waist circumference, blood pressure, lipid profiles, and plasma glucose were measured. Major cardiovascular events including cardiovascular death, myocardial infarction, stroke, and hospitalized or treated heart failure events were validated based on medical records.

**Results:**

During an average of 3.8 years of follow-up, we detected 2,076 cardiovascular events and showed that participants with depressive symptoms had an increased risk for cardiovascular events after adjustments [hazard ratio (HR): 1.29; 95% confidence index (CI): 1.08–1.53]. Stratified on metabolic risk status, the relationship between depression and cardiovascular events tended to be stronger according to the increasing numbers of metabolic risk factors, with HR (95% CI) of 0.98 (0.72–1.35) in the category with 0–2 metabolic risk factors, 1.36 (0.996–1.87) and 1.47 (1.13–1.92) for those with 3, and 4–5 metabolic risk factors, respectively, indicating an interaction effect (*P* = 0.039).

**Conclusion:**

Depression was independently associated with an increased risk of major cardiovascular events. The effect was particularly prominent among populations at higher metabolic risk.

## Introduction

Cardiovascular disease (CVD) is the leading cause of mortality worldwide (1). Numerous, but not all, studies have illuminated that major depressive disorders, and even the presence of minor depressive symptoms, increased the risk of CVD events in general populations as well as in populations with diabetes, hypertension, or coronary artery disease (2–7). Depression is the most common psychiatric disturbance, with various prevalence and prognosis in countries with different economic levels (8). Recently, China’s rapid economic development has dramatically changed the socioeconomic status, behavior, and disease spectrum of people, and also influenced their mental health. However, evidence regarding the association between depression and CVD risk was largely reported by studies conducted in Western populations; few studies have evaluated such association in Chinese populations, and with controversial findings (9–11).

Moreover, the depression status is affected by unhealthy lifestyle habits and metabolic abnormality, all of which are predominant risk factors for CVD. Previous studies have found that the population with adverse lifestyle and metabolic factors is more likely to experience depressive mood, but specific lifestyle or metabolic factors can only partially explain the increased risk of CVD attributed to depression (2, 5, 12–15). Actually, it is still unclear whether the collective status of lifestyle and metabolic risk factors may modify the association between depression and subsequent risk of major CVD events.

Therefore, based on a large-scale, population-based, nationwide, prospective cohort study of Chinese community residents, we evaluated the association between depression and major CVD events in adults, middle-aged, or older, and tested whether the relationship between depression and CVD is influenced by lifestyle or metabolic risk factors.

## Materials and Methods

### Study Design and Population

The China Cardiometabolic Disease and Cancer Cohort (4C) Study is an ongoing prospective study to investigate the association of metabolic risk factors with clinical outcomes including CVD among the Chinese population aged 40 years or older. The rationale of the 4C Study has been described in the previous publications of this study (16–19). Briefly, the baseline survey was conducted in 2011–2012, and 193,846 participants were recruited from 20 communities covering 16 provinces in Mainland China. The follow-up visit was conducted between 2014 and 2016, with an average follow-up of 3.8 years. Totally, 170,240 participants (87.8%) participated in the follow-up survey. For the current analysis, 11,681 with CVDs or 2,556 with cancer at baseline were excluded. Furthermore, 46,531 without data regarding depression status, lifestyle, and metabolic risk factors at baseline and 16,603 without verified data on incident cardiovascular events during the follow-up were excluded, leaving 92,869 participants for the current analysis. The age and sex composition of the 92,869 study participants were generally similar to those who were excluded due to missing data (Supplementary Figure 1).

The 4C Study was approved by the Ethical Review Committee of Ruijin Hospital (RUIJIN-2011-14). Written informed consent was obtained from all study participants.

### Data Collection and Definition

Data collection was conducted face-to-face at baseline and during the follow-up visit. Trained staff performed standardized questionnaires to collect information on socioeconomic status, lifestyle, and medical history. Education attainment was classified as less than high school (< 9 years) and high school or above (≥ 9 years). Marital status was classified into 3 groups: married, single, and other marital status (divorced or widowed). Dwelling status was classified into 2 groups: living alone and living with a spouse or other family members. Smoking status was classified into 2 groups: ever smoking or not. Alcohol intake was classified into two categories based on the recommendation for CVD health by sex: (1) Ideal alcohol intake: 5–29.9 g/day for men and 5–14.9 g/day for women; (2) non-ideal alcohol intake: participants who did not meet the above criteria (20). Physical activity level was classified into two categories: (1) Ideal physical activity: ≥ 150 min/week moderate intensity or ≥ 75 min/week vigorous intensity or ≥ 150 min/week moderate and vigorous intensity; (2) Physical inactivity: < 150 min/week moderate intensity or < 75 min/week vigorous intensity or < 150 min/week moderate and vigorous intensity (21). A food frequency questionnaire was used to evaluate the habitual dietary intake in the previous 12 months. The healthy diet score included the following four components: fruits and vegetables ≥ 4.5 cups/day, fish ≥ two 3.5 oz servings/week, sweets/sugar-sweetened beverages ≤ 450 kcal/week, and soy protein ≥ 25 g/day (22). An unhealthy diet was defined as a healthy diet score ≤ 1. We defined lifestyle risk status on the basis of these four lifestyle factors: unhealthy diet, physical inactivity, non-ideal alcohol intake, and current smoking, according to previous evidence in relation to CVD risk. The number of lifestyle factors was summed and classified into three groups to represent the collective lifestyle risk status: 0–1, 2, and 3–4 lifestyle risk factors.

Body weight, height, and waist circumference were measured according to a standard protocol. Three blood pressure measurements were obtained by trained observers using a calibrated automatic electronic device (OMRON Model HEM-7071) and the average of the three readings was used for the analysis. Blood samples were collected from all participants after an overnight fasting of 10–12 h. Blood glucose was measured at local hospitals using a glucose oxidase or hexokinase method within 2 h after blood sample collection under a stringent quality control program. All regional laboratories in this study have regularly participated in the proficiency-testing program and achieved the certification of external quality assessment (EQA) for glucose measurement by the National Center for Clinical Laboratories of the People’s Republic of China. All local study laboratories underwent a 5-day performance standardization process for glucose measurement. Test results were evaluated by experienced laboratory experts independently at the central laboratory, and only the local laboratories which passed the standardization program were qualified to perform study plasma glucose tests. During the study, inter-laboratory and intra-laboratory quality control assessments were conducted on each of the testing days. If the laboratory failed the internal quality control assessment, causes were identified, appropriate modifications were applied, and all blood samples were re-tested. Lipid profiles and serum creatinine were measured at the study central laboratory, which is certified by the College of American Pathologists (CAP), strictly following the quality control procedures of the laboratory. Serum samples were aliquoted into 0.5 mL Eppendorf tubes within 2 h and shipped by air in dry ice to the Study Central Laboratory at the Shanghai Institute of Endocrine and Metabolic Disease. The Laboratory has regularly participated in the proficiency-testing program and passed the Laboratory Accreditation Program of the CAP. Total cholesterol, low-density lipoprotein cholesterol, high-density lipoprotein cholesterol (HDL-C), and triglycerides (TGs) were measured using enzymatic methods with an auto-analyzer (ARCHITECT ci16200 System, Abbott Laboratories, IL, United States). Estimated glomerular filtration rate (eGFR) was calculated using the Chronic Kidney Disease Epidemiology Collaboration equation (20).

According to the widely used Adult Treatment Panel-III (ATP-III) criteria, the metabolic health status was determined based on the presence of the components of metabolic syndrome: (1) central obesity: waist circumference ≥ 90/80 cm in men or women; (2) high triglycerides: triglycerides level ≥ 1.69 mmol/L (150 mg/dL); (3) low HDL-C: HDL-C level < 1.03 mmol/L (40 mg/dL)/1.29 mmol/L (50 mg/dL) in men or women; (4) high blood pressure: blood pressure ≥ 130/85 mmHg or taking antihypertensive drugs; (5) high glycemia: fasting plasma glucose level ≥ 5.6 mmol/L (100 mg/dL) or taking hypoglycemic medications (23). The number of the metabolic syndrome components was summed and classified into three groups to represent the collective metabolic risk status: 0–2, 3, and 4–5 metabolic risk factors.

### Assessment of Depression Status

Participants underwent a screening by the Patient Health Questionnaire-9 (PHQ-9), consisting of 9 items asking about the frequency of symptoms of depression over the past 2 weeks. Each item was scored on a 0–3 scale to yield a total score ranging from 0 to 27. For the purpose of current analyses, participants who scored 5 or more were combined into one group as having the presence of depression symptom (24).

### Outcome Ascertainment

Non-fatal major CVD events including myocardial infarction, stroke, and hospitalized or treated heart failure events during the follow-up were recorded and supporting clinical documentations were obtained for blinded adjudication. Deaths and related causes were collected from local vital registries of the National Disease Surveillance Point System and National Health Insurance System. Two members of the outcome adjudication committee who were masked to the baseline characteristics of each participant independently reviewed each clinical record and verified each clinical event. Discrepancies were resolved by discussion involving other members of the committee. Incident major CVD event was defined as a composite of non-fatal myocardial infarction, non-fatal stroke, hospitalized or treated heart failure events, and cardiovascular deaths.

### Statistical Analysis

Baseline characteristics of participants according to baseline depression status were presented as means with standard deviations for continuous variables and numbers with percentages for categorical variables. Since no interactions were found between sex and depression for the risk of CVD events (*P* for interaction = 0.19), pooled analyses were presented. In the time-to-event analysis, participants were censored at the date of CVD diagnosis, death, or the end of follow-up, whichever occurred first. Person-time was calculated from the enrollment date to the censoring date for each participant. Missing data for risk factor variables were deleted from the analyses.

Association of depression status with the risk of incident major CVD events was evaluated using Cox proportional hazards models with adjustment for potential confounding factors including age, sex, urban/rural residence, economic status of study site, body mass index (BMI), education attainment, marital status, dwelling status, lifestyle, and metabolic risk factors. Furthermore, we combined the depression status with each lifestyle or metabolic risk factor to detect the joint effect of depression status and other modifiable factors on major CVD events.

To test whether the collective status of lifestyle or metabolic risk factors may modify the association between depression and subsequent risk of major CVD events, the numbers of lifestyle or metabolic risk factors were summed for each participant to represent an overall risk status, and were further classified into three categories (lifestyle risk factors: 0–1, 2, and 3–4 risk factors; metabolic risk factors: 0–2, 3, and 4–5 risk factors). We evaluated the association of depression status with incident CVD events according to lifestyle or metabolic risk status and tested the multiplicative interaction between depression and risk status by including the respective interaction term in the model (for example, depression × lifestyle risk status), with the main effect included in the models as well.

All reported *P*-values are nominal and two sided. We used SAS software, version 9.2, for statistical analyses.

## Results

Baseline characteristics of the study population are presented in Table 1. Among 92,869 participants aged less than or equal to 40 years recruited from communities across Mainland China, 5,576 individuals (6.0%) had depression symptom as categorized by a high baseline depression score (PHQ-9 ≥ 5). Compared with those without depression symptom, participants who had depression symptom were more likely to be female, to be single, divorced, or widow, to be living alone and had high school or above education. The depression group had a lower proportion of current cigarette smokers and participants with physical inactivity. In addition, participants with the presence of depression symptom tended to have a lower level of BMI, waist circumference, and blood pressures. The proportion of participants with family history of CVD was markedly higher among those with depression symptom. Levels of other lifestyle and metabolic risk factors including unhealthy diet, alcohol intake, fasting plasma glucose, total cholesterol, low-density lipoprotein cholesterol (LDL-C), HDL-C, triglycerides, and eGFR did not significantly differ between two groups according to the depression status.

**TABLE 1:** Baseline characteristics of the study population according to the baseline depression status.

	Depression symptom		*P*-value
	None	Presence	
No. of participants	87,293	5,576	–
Age at baseline, year	56.2 ± 8.9	56.3 ± 8.5	0.57
Male gender, no. (%)	29,929 (34.3)	1,322 (23.7)	< 0.0001
BMI, kg/m^2^	24.7 ± 3.6	24.3 ± 3.6	< 0.0001
High school or above education, no. (%)	34,449 (39.5)	2,530 (45.4)	< 0.0001
Married, no. (%)	80,135 (91.8)	4,805 (86.2)	< 0.0001
Living alone, no. (%)	3,024 (3.5)	403 (7.2)	< 0.0001
Family history of CVD, no. (%)	14,819 (17.0)	1,295 (23.2)	< 0.0001
Unhealthy diet, no. (%)	30,380 (34.8)	1,964 (35.2)	0.52
Physical inactivity, no. (%)	74,735 (85.6)	4,671 (83.8)	0.0001
Ideal alcohol intake, no. (%)	4,128 (4.7)	260 (4.6)	0.82
Ever smoking, no. (%)	17,231 (19.7)	923 (16.6)	< 0.0001
Waist circumference, cm	84.3 ± 9.7	83.7 ± 10.0	< 0.0001
Systolic blood pressure, mmHg	131.2 ± 20.0	128.3 ± 20.2	< 0.0001
Diastolic blood pressure, mmHg	77.9 ± 10.9	76.7 ± 11.0	< 0.0001
Fasting plasma glucose, mg/dl	107.5 ± 28.9	108.1 ± 31.1	0.099
Triglycerides, mg/dl	116.9 (83.3–171.0)	117.8 (83.3–170.1)	0.61
LDL-C, mg/dl	109.9 ± 33.8	110.0 ± 34.7	0.91
HDL-C, mg/dl	50.8 ± 13.5	50.9 ± 13.7	0.37
Total cholesterol, mg/dl	189.4 ± 44.0	189.4 ± 45.2	0.95
eGFR, ml/min/1.73 m^2^	96.2 (87.1–103.1)	96.4 (86.9–102.8)	0.93

*BMI, body mass index; CVD, cardiovascular disease; LDL-C, low-density lipoprotein cholesterol; HDL-C, high-density lipoprotein cholesterol; eGFR, estimated glomerular filtration rate. Values are numbers (proportions), means ± standard deviations, or medians (interquartile ranges). To convert the values of cholesterol to millimoles per liter, multiply by 0.02586. To convert the values of triglycerides to millimoles per liter, multiply by 0.01129. To convert the values of glucose to millimoles per liter, multiply by 0.05551.*

A total of 2,076 participants in the current data set developed major CVD events including non-fatal myocardial infarction, non-fatal stroke, hospitalized or treated heart failure, and cardiovascular deaths during an average of 3.8 years follow-up. Table 2 shows that the incidence rate of major CVD events was significantly higher in participants with depression symptoms (148 cases, 2.65%, 7.35/1,000 person-years), compared with those without depression symptoms (1,928 cases, 2.21%, 6.18/1,000 person-years), with a *P*-value of 0.029. Cox proportional hazards models showed that participants with depression symptoms had significantly increased risk for major CVD events after sufficient adjustments for age, sex, urban/rural resident, economic status, family history of CVD, socioeconomic factors, lifestyle risk factors, and metabolic risk factors [the multivariable adjusted hazard ratio (HR): 1.29; 95% confidence index (CI): 1.08–1.53; *P* = 0.004] (Table 2).

**TABLE 2:** Association between depression status and major CVD events.

	Depression symptom		*P*-value
	None	Presence	
No. of participants	87,293	5,576	–
Person-years	311,749	20,135	–
Cases (%)	1,928 (2.21)	148 (2.65)	0.029
Incidence rate,/1,000 person-years	6.18	7.35	0.029
Model 1, HR (95% CI)	1.00 (ref.)	1.28 (1.08–1.51)	0.004
Model 2, HR (95% CI)	1.00 (ref.)	1.28 (1.08–1.51)	0.005
Model 3, HR (95% CI)	1.00 (ref.)	1.27 (1.07–1.51)	0.006
Model 4, HR (95% CI)	1.00 (ref.)	1.29 (1.08–1.53)	0.004

*Model 1: Adjusted for age, sex, urban/rural residence, and economic status. Model 2: Further adjusted for education attainment (below high school, high school, or above), marriage status (married, single, divorced, or widow), dwelling status (living alone or not), family history of CVD (yes or no), and BMI based on Model 1. Model 3: Further adjusted for lifestyle risk factors including unhealthy diet (yes or no), physical inactivity (yes or no), alcohol intake (non-ideal or ideal), and ever smoking (yes or no) based on Model 2. Model 4: Further adjusted for metabolic risk factors including central obesity (yes or no), high triglycerides (yes or no), low HDL-C (yes or no), high blood pressure (yes or no), and high glycemia (yes or no) based on Model 3. CVD, cardiovascular disease; HR, hazard ratio; CI, confidence index; HDL, high density lipoprotein cholesterol.*

**FIGURE 1:**
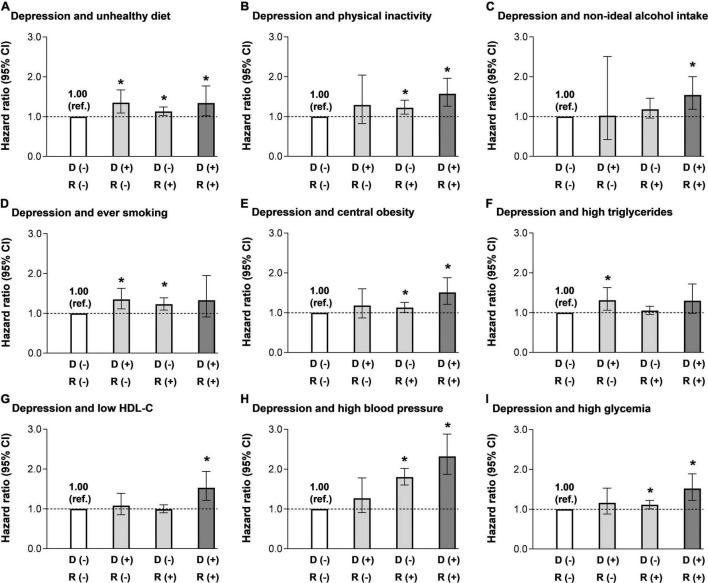
Multivariable-adjusted hazard ratios (HRs) [95% confidence indices (CIs)] for major cardiovascular disease (CVD) events according to the combination of depression and other lifestyle and metabolic risk factors. **(A)** Groups according to the combination of depression and unhealthy diet. **(B)** Groups according to the combination of depression and physical inactivity. **(C)** Groups according to the combination of depression and alcohol intake. **(D)** Groups according to the combination of depression and smoking status. **(E)** Groups according to the combination of depression and central obesity. **(F)** Groups according to the combination of depression and high triglycerides. **(G)** Groups according to the combination of depression and low high-density lipoprotein cholesterol (HDL-C). **(H)** Groups according to the combination of depression and high blood pressure. **(I)** Groups according to the combination of depression and high glycemia. Adjusted for age, sex, education attainment (below high school, high school, or above), marriage status (married, single, divorced, or widow), dwelling status (living alone, or not), family history of CVD (yes or no), and body mass index (BMI). Individual lifestyle and metabolic risk factors were mutually adjusted. D (–): without depression symptom; D (+): with depression symptom; R (–): without the specific combined risk factor; R (+): with the specific combined risk factor. *P < 0.05 compared with the reference group.

For individual lifestyle risk factor, unhealthy diet, physical inactivity, and current smoking showed significant associations with major CVD events, with a multivariate-adjusted HR of 1.12 (95% CI: 1.02–1.23) for unhealthy diet, 1.22 (95% CI 1.07–1.40) for physical inactivity, and 1.21 (95% CI: 1.07–1.37) for ever smoking, respectively. As to individual metabolic risk factor, analyses indicated that the risk increased significantly for participants with central obesity, high blood pressure, and high glycemia for major CVD events, with a multivariate-adjusted HR of 1.14 (95% CI: 1.02–1.27) for central obesity, 1.80 (95% CI: 1.61–2.01) for high blood pressure, and 1.12 (95% CI: 1.02–1.23) for high glycemia, respectively (Supplementary Table 1). When combining depression status with individual lifestyle or metabolic risk factor, associations were generally stronger in participants who were exposed to both depression and specific lifestyle or metabolic risk factors (Figure 1). Nevertheless, compared with never-smokers without depression (considered as a control group), never-smokers with depression or ever smokers without depression symptoms showed significantly higher HRs for major CVD events [1.35 (95% CI: 1.11–1.63) for never-smokers with depression, and 1.23 (95% CI: 1.08–1.39) for ever smokers without depression], whereas no significant increased risk was detected for subjects with both depression and ever smoking (HR: 1.33; 95% CI: 0.91–1.95) (Figure 1D). In the combined analysis of depression and high triglycerides, only subjects with depression but without high triglycerides showed an increased risk for major CVD events (Figure 1F).

**FIGURE 2:**
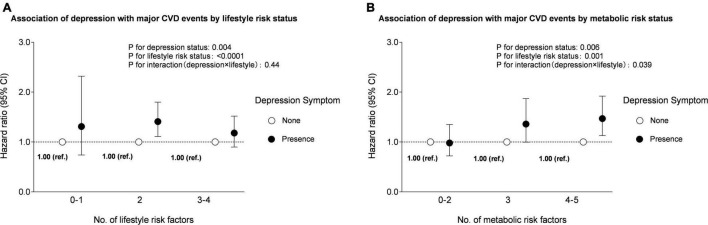
Multivariable-adjusted HRs (95% CIs) for major CVD events according to the combination of depression with lifestyle and metabolic risk status. **(A)** Groups according to the combination of depression and lifestyle risk status. Adjusted for age, sex, education attainment (below high school, high school or above), marriage status (married, single, divorced, or widow), dwelling status (living alone, or not), family history of CVD (yes or no), BMI, central obesity (yes or no), high triglycerides (yes or no), low HDL-C (yes or no), high blood pressure (yes or no), and high glycemia (yes or no). **(B)** Groups according to the combination of depression and metabolic risk status. Adjusted for age, sex, education attainment (below high school, high school, or above), marriage status (married, single, divorced, or widow), dwelling status (living alone, or not), family history of CVD (yes or no), BMI, unhealthy diet (yes or no), physical inactivity (yes or no), alcohol intake (non-ideal or ideal), and ever smoking (yes or no).

Association of depression status with major CVD events were stratified by categories according to the number of lifestyle risk factors (Figure 2A) or metabolic risk factors (Figure 2B). In particular, the presence of depression symptom was associated independently and significantly with the risk for major CVD events only in participants with medium lifestyle risk. The adjusted HR for the presence of depression symptom was 1.31 (0.74–2.32) in the individuals with 0–1 lifestyle risk factor, 1.41 (1.11–1.8076), and 1.18 (0.90–1.52) for those with 2, and 3–4 lifestyle risk factors, respectively. With regard to subgroups, according to the number of metabolic risk factors, the risk related to the presence of depression symptom tended to be stronger as the metabolic risk status deteriorates. The adjusted HR was 0.98 (0.72–1.35) in the category with 0–2 metabolic risk factors, 1.36 (0.996–1.87) and 1.47 (1.13–1.92) for those with 3, and 4–5 metabolic risk factors, respectively. A significant interaction was observed for depression and metabolic risk status in the association with major CVD events (*P* for interaction: 0.039).

## Discussion

We found a significant and independent association between depression and incident major CVD events in a population-based prospective cohort. Importantly, we reported that the CVD risk associated with depression was stronger among population with adverse metabolic risk status, as well as several lifestyle risk factors. Our findings underline the importance of the recognition and treatment of depression, especially for those with unhealthy lifestyle and metabolic abnormality. To our knowledge, this is the first and the largest epidemiological study which comprehensively investigated the combined effects of depression status and the overall lifestyle and metabolic status on major CVD events.

Previous studies have demonstrated a significant association of depression with CVD risk, mainly in the Western populations. Findings from a meta-analysis of 30 prospective studies reported a 30% of higher risk of coronary heart disease and myocardial infarction, and indicated a stronger association between coronary heart disease and depression for participants from the United States than for participants from Europe; whereas no statistically significant risk was detected in population from Hong Kong (9). The Prospective Urban Rural Epidemiology (PURE) study, conducted in 21 countries, reported that depression was associated with CVD in middle-income countries, but not on high- or low-income countries (8). Apparently, the association between depression and CVD risk differed by ethnicity and regions due to various social structure, economic levels, and cultural background. However, studies from China on the effect of depression on CVD risk were scarce and provided tentative evidence. Very recently, the China Kadoorie Biobank (CKB) study suggested that participants with major depression had an increased risk of ischemic heart disease (HR: 1.32; 95% CI 1.15–1.53) and provided the first prospective, population-based evidence in Mainland China (10). In addition, using data from the CKB study and Dongfeng-Tongji (DFTJ) study, depression was recognized to be associated with an elevated risk of all-cause and CVD mortality (11). Our results from a large prospective nationwide cohort presented an independently significant risk of depression symptom, which were in line with the latest study that further extended the findings by the combinations of lifestyle and metabolic risk factors, and determined the joint effects of coexistence of depression and lifestyle and metabolic status on CVD risk.

Several studies have investigated the modification effect of individual conventional cardiovascular risk factors on the association between depression and CVD risk and suggested that unfavorable prognosis in patients with depression can partly be mediated by some unhealthy habits, such as smoking, physical inactivity, and poor nutrition (2, 5, 13, 15, 25). Moreover, stratified analysis demonstrated that the association between depression and CVD risk was stronger in patients with diabetics (4). However, the effect of depression with combined lifestyle and metabolic risk status on CVD risk is not conclusive. In the current analysis, we focused on nine modifiable conventional cardiovascular risk factors, including four lifestyle and five metabolic risk factors. Participants with both depression and comorbid conventional risk factor had worse prognosis than those having either depression or comorbid conventional risk factor. Particularly, we found that depression, together with physical inactivity conferred higher risk of major CVD events, which was in line with a previous finding that suggests that depression could reduce the effectiveness of physical activity in the prevention of cardiometabolic disorders and added a new piece of evidence to particularly target ideal physical activity in people who experience depression to attenuate CVD risk (26). Moreover, we found a significantly larger magnitude of the association for depression and major CVD events in participants with higher metabolic risk, and identified a synergistic effect between metabolic risk status and depression on CVD risk by presenting a significant interaction. Our findings suggest the need for a multi-pronged approach to manage psychological, lifestyle, and metabolic risk in the prevention of CVD, and particularly highlight the importance of evaluation and promotion of psychological health, especially in people who have higher lifestyle and metabolic risk.

According to previous findings, a potential explanation of the association between depression and CVD risk would be the probability that people with unhealthy lifestyle habits or with metabolic disorders were vulnerable to depression. Although our results indicated that participants with both depression symptom and specific lifestyle or metabolic risk coffered a higher risk of major CVD events, data from our baseline characteristics showed that subjects with depression even had a slightly lower proportion of physical inactivity and smoking, as well as lower level of blood pressure, BMI, and waist circumference. In addition, there is no statistical difference in unhealthy diet, drinking status, and levels of lipids and glucose across depression status. The findings that worse metabolic spectrum was not detected in participants with depression appears to be partly explained by the fact that we evaluated the depression status and metabolic risk factors simultaneously, and majority of the participants were unaware of their metabolic abnormality. Meanwhile, compared to Western cultures, being little overweight, elderly individuals, particularly in in Asian cultures, have not been regarded as unhealthy, which are coincident with previous studies in Chinese and Korean populations. This could be considered as supporting the “Jolly Fat” hypothesis (11, 27–29). On the other hand, we found that the proportion of participants with family history of CVD was markedly higher among those with depression symptom, which also suggested that the awareness of higher risk for cardiometabolic disease is related to the presence of depression.

The main strengths of this study included a large sample and the combination of a comprehensive evaluation of lifestyle and clinical covariates assessed by standardized measurements. Various limitations have to be addressed. First, the follow-up duration was relatively short with a mean of 3.8 years, which would limit the number of CVD events and make it difficult to conduct further stratified analysis according to age, sex, or family history of CVD. However, no significant interaction effect of depression with age, sex, and family history of CVD on CVD events was detected; and these variables and a wide spectrum of covariates was included in the adjustment to rule out the confounding effect. Second, the depression status was defined according to a screening instrument by a cut-off of PHQ-9 score ≥ 5 to indicate the presence of depression symptoms, rather than the clinical diagnosis of major depression episode. However, the PHQ-9 can comprehensively assess each of the nine domains that define depression and is a highly recommended tool for screening to reflect early stage depression in general population and in patients at the high risk of CVD (30).

In summary, depression was independently associated with an increased risk of CVD events in a large national cohort of Chinese population aged ≥ 40 years. More importantly, the effect of depression on CVD risk was stronger among population with conventional metabolic risk factors. Our findings highlight the importance of depression screening and management for the prevention of CVD events, as well as the modification of lifestyle habits and metabolic conditions.

## Data Availability Statement

The raw data supporting the conclusions of this article will be made available by the authors, without undue reservation.

## Ethics Statement

The studies involving human participants were reviewed and approved by the Ethical Review Committee of Ruijin Hospital (RUIJIN-2011-14). The patients/participants provided their written informed consent to participate in this study.

## Author Contributions

XC, ZhL, and YY contributed to the analysis of data and drafted the manuscript. GC, QW, GQ, LY, GW, YQ, ZuL, XT, YH, RH, ZY, LS, ZG, QS, YM, JZ, LuC, TZ, QL, FS, LiC, YZ, YW, HD, CL, SW, TY, ML, YX, MX, TW, ZZ, JL, and YB contributed to data acquisition, analysis and interpretation. XY, WW, and GN contributed to the conception and design, and critically revised the manuscript. All authors read and approved the final manuscript.

## Conflict of Interest

The authors declare that the research was conducted in the absence of any commercial or financial relationships that could be construed as a potential conflict of interest.

## Publisher’s Note

All claims expressed in this article are solely those of the authors and do not necessarily represent those of their affiliated organizations, or those of the publisher, the editors and the reviewers. Any product that may be evaluated in this article, or claim that may be made by its manufacturer, is not guaranteed or endorsed by the publisher.
