# Supplementary material for: Depression Status, Lifestyle, and Metabolic Factors With Subsequent Risk for Major Cardiovascular Events: The China Cardiometabolic Disease and Cancer Cohort (4C) Study
Source: Front Cardiovasc Med. 2022 May 26;9:865063. doi: 10.3389/fcvm.2022.865063 (PMC9177939; doi:10.3389/fcvm.2022.865063)
Supplement: Supplementary file 1 [file Data_Sheet_1.doc]

***Supplementary Materials***

**1 Supplementary Tables**

**Supplementary Table S1.** Associations of lifestyle and metabolic risk factors with major CVD events

|  | Person-years | Cases | HR (95% CI)† |
| --- | --- | --- | --- |
| Unhealthy diet |  |  |  |
| No | 217,599 | 1,274 | 1.00 (ref.) |
| Yes | 114,286 | 802 | 1.12 (1.02-1.23) |
| Physical inactivity |  |  |  |
| No | 48,343 | 245 | 1.00 (ref.) |
| Yes | 283,541 | 1,831 | 1.22 (1.07-1.40) |
| Non-ideal alcohol intake |  |  |  |
| No | 15,623 | 104 | 1.00 (ref.) |
| Yes | 316,261 | 1,972 | 1.20 (0.98-1.47) |
| Ever smoking |  |  |  |
| No | 267,046 | 1,552 | 1.00 (ref.) |
| Yes | 64,838 | 524 | 1.21 (1.07-1.37) |
| Central obesity |  |  |  |
| No | 149,654 | 775 | 1.00 (ref.) |
| Yes | 182,230 | 1,301 | 1.14 (1.02-1.27) |
| High triglycerides |  |  |  |
| No | 223,843 | 1,305 | 1.00 (ref.) |
| Yes | 108,042 | 771 | 1.04 (0.95-1.15) |
| Low HDL-C |  |  |  |
| No | 201,257 | 1,258 | 1.00 (ref.) |
| Yes | 130,627 | 818 | 1.02 (0.93-1.12) |
| High blood pressure |  |  |  |
| No | 136,419 | 440 | 1.00 (ref.) |
| Yes | 195,465 | 1,636 | 1.80 (1.61-2.01) |
| High glycemia |  |  |  |
| No | 170,454 | 837 | 1.00 (ref.) |
| Yes | 161,430 | 1,239 | 1.12 (1.02-1.23) |

Adjusted for age, sex, education attainment (below high school, high school or above), marriage status (married, single, divorced or widow), dwelling status (living alone, or not), family history of CVD (yes or no), BMI, and depression symptoms (none or presence). Individual lifestyle and metabolic risk factors were mutually adjusted.

Abbreviations: CVD, cardiovascular disease; HDL-C, high density lipoprotein cholesterol; BMI, body mass index.

**2 Supplementary Figures**


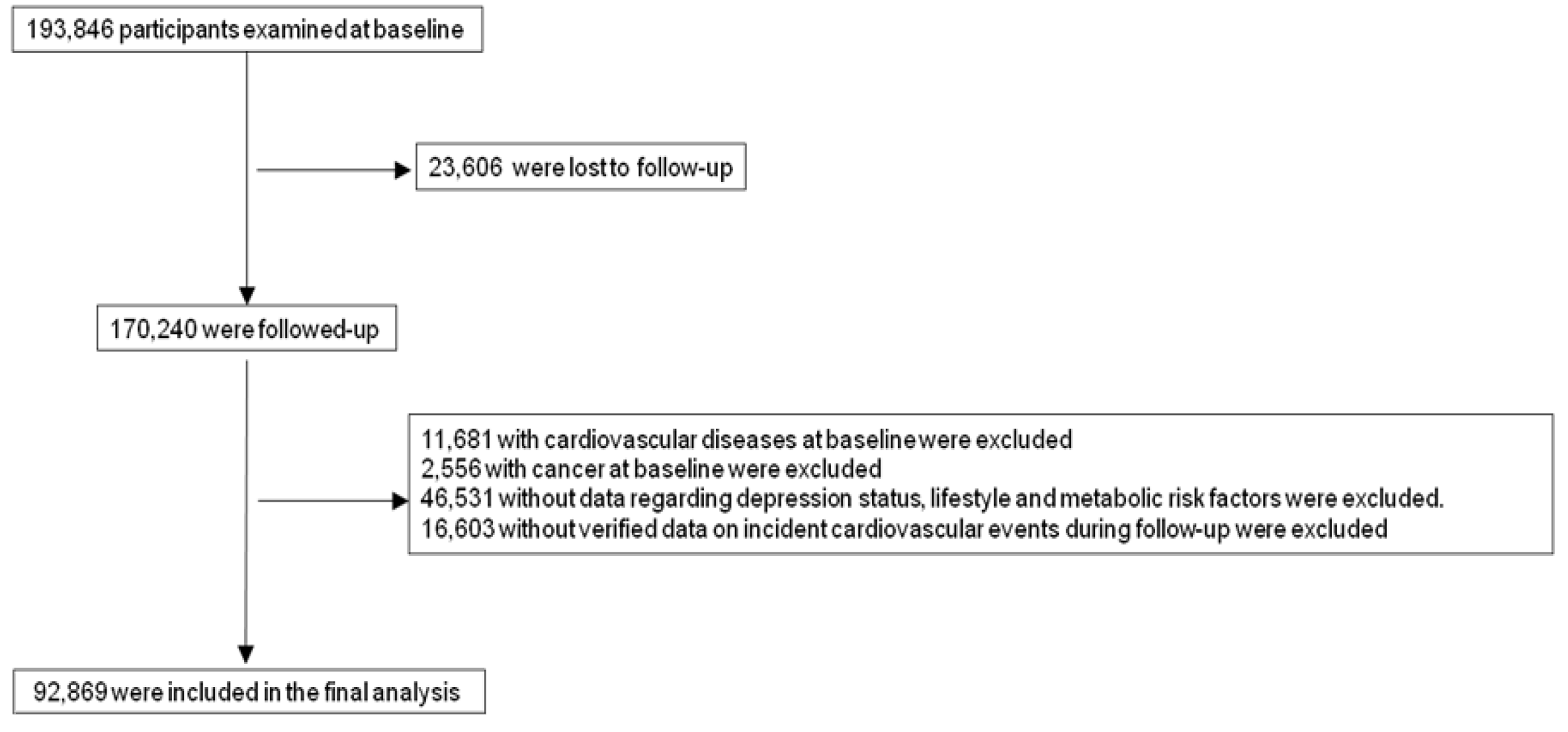


**Supplementary Figure S1.** Flowchart of the analysis.
